# Occupational noise and age: A longitudinal study of hearing sensitivity as a function of noise exposure and age in South African gold mine workers

**DOI:** 10.4102/sajcd.v67i2.687

**Published:** 2020-03-17

**Authors:** Leoni M. Grobler, De Wet Swanepoel, Susan Strauss, Piet Becker, Zahan Eloff

**Affiliations:** 1 Department of Speech-Language Pathology and Audiology, Faculty of Humanities, University of Pretoria, Pretoria, South Africa; 2 Department of Speech-Language Therapy and Audiology, Faculty of Humanities, University of Pretoria, Pretoria, South Africa; 3 Department of Health Sciences, Faculty of Health, University of Pretoria, Pretoria, South Africa; 4 Occupational Health Department, AngloGold Ashanti, Carletonville, South Africa

**Keywords:** Age-related hearing loss, Noise exposure, Noise-induced hearing loss, Occupational noise exposure, Mining

## Abstract

**Background:**

A relationship exists between occupational noise exposure and age, which remains poorly understood.

**Objectives:**

The aim of this study was to establish the relationship between hearing loss and age over time.

**Method:**

Audiological data from 2583 mine workers in South Africa were utilised. Data were received from a non-noise exposed group (NNEG) (*n* = 951) and a noise exposed group (NEG) (≥85 dBA) (*n* = 1632). Data comprised a low-frequency average (LFA512) (average of audiological thresholds for 0.5 kHz, 1 kHz and 2 kHz) and high-frequency average (HFA346) (average of audiological thresholds for 3 kHz, 4 kHz and 6 kHz). Data were compared by using mixed-effects regression analysis.

**Results:**

Base threshold values were higher for the NEG than for the NNEG across frequencies. All year-to-year increases in mean hearing thresholds were statistically significant (*p* < 0.01). When correcting for age, increases in mean hearing thresholds were higher for the NEG than for the NNEG for HFA346 (3.5 dB vs. 2.9 dB decline over a 4-year period) but similar for LFA512 (0.6 dB vs. 0.7 dB decline). Uncorrected for age, increases in mean hearing thresholds were higher than when age was corrected for.

**Conclusion:**

Age and occupational noise exposure influence hearing thresholds over time. The continued increase in hearing thresholds of the NEG above that of the NNEG can be related to ineffective noise management programmes and/or the fact that early noise exposure leads to a higher burden of hearing loss over time – even after noise exposure had stopped.

## Introduction

Excessive noise is an occupational hazard with many adverse effects, including elevated blood pressure (Kerns, Masterson, Themann, & Calvert, 2018; Wang et al., 2018), elevated cholesterol (Kerns et al., 2018), increased risk for coronary heart disease (Eriksson et al., 2018), reduced work performance, difficulty sleeping, annoyance, stress, tinnitus, temporary threshold shift (TTS) and noise-induced hearing loss (NIHL) (Nelson, Nelson, Concha-Barrientos, & Fingerhut, 2005). Although many other adverse effects of high-level noise exposure have been reported, NIHL is recognised as the primary and most direct health effect of noise exposure, which makes it a significant and ongoing health concern with economic consequences (Agrawal, Niparko, & Dobie, 2010; Hong, Kerr, Poling, & Dhar, 2013; Lie et al., 2016).

Noise-induced hearing loss is a significant health concern as it constitutes a permanent shift in hearing thresholds caused by prolonged exposure to high levels of noise, as a direct cause of damage to the sensory hair cells of the inner ear (Sliwinska-Kowalska & Davis, 2012). A study based on data from the United States of America (USA) estimated that the industry with the highest proportion of workers exposed to hazardous noise is mining, with an estimated 85% of production workers and labourers exposed to noise levels above 85 dB (Time-weighted Average (TWA) (Nelson et al., 2005). These results are consistent with the results from similar studies conducted in the USA and Scandinavia (Engdahl & Tambs, 2010; Masterson, Deddens, Themann, Bertke, & Calvert, 2015; Tak & Calvert, 2008). Statistics regarding the prevalence of occupational NIHL in developing countries are not readily available, (Nelson et al., 2005); however, according to Chadambuka, Mususa and Muteti (2003), 80% of individuals affected by occupational NIHL reside in low- and middle-income countries, such as South Africa. In 2007, it was estimated that nearly half of the mining industry’s workforce in South Africa was exposed to hazardous occupational noise, and of these individuals, more than 90% work in zones where noise level exceeds the 85 A-weighted decibels (dBA) time-weighted average, with 11% working in areas in which the noise levels are even more hazardous (Hermanus, 2007). In 2011, Edwards, Dekker and Franz (2011) reported that approximately 73.2% of the mine employees in South Africa were exposed to noise levels above the legislated occupational exposure level of 85 dBA. Noise-induced hearing loss follows only age-related hearing loss (ARHL) as the largest contributor to hearing loss globally. Therefore, most hearing losses not associated with noise can be attributed to age (Lie et al., 2016).

Both ARHL and NIHL in humans are multifactorial, with contributions from and interactions between numerous intrinsic as well as extrinsic variables that can shape their final outcome (Kujawa & Liberman, 2006; Yamasoba et al., 2013). The interaction between age and noise and their respective effects on hearing status of noise-exposed individuals is still poorly understood, making it difficult to distinguish between NIHL and ARHL at an individual level, and often because the time and magnitude of noise exposure are unclear (Basner et al., 2014; Gates, Schmid, Kujawa, Nam, & D’Agostino, 2000; Rosenhall, 2003; Xiong, Yang, Lai, & Wang, 2013). Numerous studies have examined the interaction between NIHL and ARHL, but because these conditions occur concurrently, it is difficult to determine their individual effects on hearing (Aarhus, Tambs, Nafstad, Bjorgan, & Engdahl, 2016; Gates et al., 2000; Rosenhall, 2003; Xiong et al., 2013).

In recent attempts to explain these individual effects and interactions between NIHL and ARHL, two possible models of interaction have emerged (Aarhus et al., 2016): super additive versus less than additive interaction. A possible super additive interaction follows the notion that an already damaged hair cell is more susceptible to further damage from additional factors. When describing the interaction between NIHL and ARHL as a less than additive effect, the assumption is based on the premise that when a number of hair cells were previously damaged, there is a decreased risk of further damage. Simply stated, hair cells lost from one cause cannot be lost again from a second cause (Dobie, 2008; Gallo & Glorong, 1964; Gates et al., 2000).

It is known that hair cell damage is a key contributor to NIHL and ARHL and therefore most evidence gathered with regard to the interaction between NIHL and ARHL is based on audiometric thresholds as defined by the audiogram, which measures the minimum sound pressure level required for pure tone detection in quiet conditions (Fernandez, Jeffers, Lall, Libberman, & Kujawa, 2015; Liberman, 2017). For years, it has been assumed that cochlear neural loss manifested only after hair cell loss and was rarely seen as significant in NIHL and ARHL (Liberman, 2017). Recent research however demonstrates that in both NIHL and ARHL, synaptic connections between the hair cells and cochlear neurons can be destroyed even before hair cell damage occurs (Liberman, 2017). This challenges the traditional view held by various researchers (Dias, Cordeiro, Corente, & Concalves, 2006; Dobie, 2008; Jonsson & Rosenhall, 1998; Rosenhall, 2003; Teles & Medeiros, 2007) that the influence of noise on hearing is time limited, implying that the progression of NIHL ceases when noise exposure stops.

In view of the above, the rationale of this study was to measure the decline in hearing thresholds in noise-exposed individuals, as well as non-noise-exposed individuals over time, in order to establish if early noise exposure leads to a larger burden of hearing loss in later life as an individual ages. This was done by developing a regression model for the prediction of hearing loss as a function of noise exposure and age, over time, in a large longitudinal data set of gold miners in South Africa.

## Methods

A retrospective cohort design was followed. Data for the study were obtained from the occupational health departments in the AngloGold Ashanti group of gold mines in South Africa, the world’s third largest gold mining company when measured by production (Anglo Gold Ashanti, 2018). Data for the study included demographic and audiological information of participants between 2001 and 2008.

The participants in this study were employees at two gold mine groups, consisting of seven gold mines within the AngloGold Ashanti group. The data set included audiological, biographical and environmental information. Records included data for 57 714 employees, comprising a total of 232 458 audiograms. Every employee had at least a baseline audiogram and an annual audiogram. All participants above 18 years across all genders and cultural groups were included. All data were collected between 2001 and 2008 from routine audiological screening, as well as diagnostic follow-up measures, made available by the mine’s occupational health department. Available audiological thresholds comprised the following frequencies: 0.5 kHz, 1 kHz, 2 kHz, 3 kHz, 4 kHz, 6 kHz and 8 kHz. Employees were further defined in terms of specific noise exposure levels, based on noise measurements made available from the mine’s noise hygienist. Within these noise categories, specific occupations were used to further classify employees (e.g. rock driller or administration worker).

Audiological testing was conducted between 2001 and 2008 in sound-treated rooms that complied with the requirements as stipulated in the South African National Standards (SANS) document (SANS 10154-1:^2001^). A Tremetrics RA600 Type 4 audiometer was used for testing, coupled with TDH39 headphones. Ten workers can be tested simultaneously using the automatic testing setting of the Tremetrics audiometer. From here, audiological information is automatically updated to a database on software specifically designed for this purpose (Everest). Follow-up diagnostic audiometry was performed on employees whose percentage loss of hearing (PLH) had dropped by more than 10% from their baseline test by using a GSI 61 diagnostic audiometer. All diagnostic test results were also captured with the Everest software. Screening and diagnostic audiometers had valid calibration certificates at the time of testing. The Everest database was made available for research purposes in 2012, and utilised by Strauss (2012). The current study is a follow-up of the original study.

The *Mine Health and Safety Act*, instruction 171 (Republic of South Africa, 2001), and the South African National Standard (SANS 10083:^2013^) require that all employees with noise exposure of 85 dBA normalised in an 8-h working day or 40-h working week should be monitored audiologically. Legislation as set out in instruction 171 (Republic of South Africa, 2001) makes it compulsory for all employees to have a baseline audiogram within 2 years of the legislation being passed (2001), or within 30 days of employment for new employees. AngloGold Ashanti complied with these regulations, and therefore audiograms from 2001 onwards were available within this data set. All data collected complied with this guideline; audiograms consisted of baseline, annual screening and exit audiograms that were recorded by occupational health personnel. Results of diagnostic testing were recorded by audiologists registered with the Health Professional Council of South Africa. Diagnostic audiograms were noted for all employees where their PLH values exceeded their baseline audiogram by 10%, as regulated by instruction 171 (Republic of South Africa, 2001). Where PLH was under 10%, only baseline, screening and exit results were used. Audiological data comprised thresholds in dB HL for the following frequencies: 0.5 kHz, 1 kHz, 2 kHz, 3 kHz, 4 kHz, 6 kHz and 8 kHz, for both the left and right ears. The left and right ear values were averaged and then, using these values, an average was worked out for the low frequencies (0.5 kHz, 1 kHz and 2 kHz) referred to as low-frequency averages (LFA), and high frequencies (3 kHz, 4 kHz and 6 kHz), referred to as high-frequency average (HFA). High frequencies and low frequencies were separated, as both noise and age have a greater influence on higher frequencies (Dias et al., 2006; Teles & Medeiros, 2007). Where screening and diagnostic results were available, diagnostic results were used.

Apart from the above, age of employee, occupation (classified according to noise exposure level), years of service, race and gender were also gathered from the database. Data were exported from the mine’s electronic database (Everest) into Microsoft Excel for data processing.

Employees were divided into four noise exposure groups, namely, above surface noise exposure (≥ 85 dBA), below surface noise exposure (≥ 85 dBA), no noise exposure and uncertain noise exposure. In order to have homogeneous exposure groups for comparison in this study, two subgroups from the below surface noise exposure group and the no-noise exposure group were selected: namely, the Rock Drillers (NEG) and Administration Workers (NNEG). The Rock Drillers were chosen because noise exposure in mining is mainly because of the use of heavy equipment, drilling and rock breaking, transferring, sorting and milling of rock and the confined working environment (Hermanus, 2007). In a study conducted in South Africa in 2007, the mean noise levels of four commonly used drills (self-propelled drill, pneumatic drill, hydraulic drill and electrical drill) were measured between 84.9 dBA and 107.9 dBA (Phillips, Heyns, & Nelson, 2007). These levels fall close to or above the maximum defined occupational noise exposure of ≥ 85 dBA, as classified according to the South African regulations on the daily permissible level of noise exposure (Republic of South Africa, 2001).

Data received included records for 4399 rock drillers and 2211 administration personnel. For this study, only data where employees had four longitudinal (annual) audiograms, falling in sequence anywhere between 2001 and 2008 (e.g. 2001, 2002, 2003 and 2004, or 2003, 2004, 2005 and 2006), were used in order to be able to perform a mixed-effects regression analysis. For this, raw data were exported to a sequential query language (SQL) database where the data were segregated into islands of serial data, and thereafter exported back into Microsoft Excel for statistical analysis. Four serial audiograms were utilised as this was enough to measure an increase in hearing thresholds over time, while still keeping the sample size representative of the bigger population. This reduced the total participant number to 2583 employees. This included 1632 in the noise-exposed (rock driller) group, and 951 in the non-noise-exposed (administration worker) group. Participants’ age, race and gender are presented in Table 1. Participants’ ages ranged from 19 to 61 years. The NEG were on average 7.1 years older than the NNEG. The majority of the participants were black males. According to the 2011 South African census, 79.4% of the population identifies themselves as black people (Statistics South Africa, 2019), as opposed to the sample where approximately 98% of participants were blacks. After topping 14% in early 2015, women representation in South Africa’s mining industry is now 10% (International Woman in Mining, 2019), which is higher than the 0.6% women represented in this sample. In total, the sample represented 22% of all original data received. The external validity of the study will be limited to the gold mining sector within South Africa, as other factors such as migrant living conditions, prevalent health co-morbidities and social lifestyle will be unique to the population sampled. However, as the entire population of the seven gold mines that partook in this study is represented in the sample, conclusions reached can reliably represent these specific mines.

**TABLE 1:** Age, race and gender distribution of the 2583 gold mine workers.

Age (at first test)	Mean	Min age	Max age	Gender				Race			
				Female		Male		White		Black	
				*n*	*%*	*n*	*%*	*n*	*%*	*n*	*%*
Non-noise	36.2	20	61	9	52.9	942	36.7	38	80.9	913	36
Noise	43.3	19	60	8	47.1	1624	63.3	9	19.1	1623	64
**Total**	-	-	-	**17**	**100**	**2566**	**100**	**47**	**1000**	**2536**	**100**

### Data analysis

A mixed-effects regression analysis was employed for the data where four longitudinal (yearly) audiograms were available for an individual in the cohort. A mixed-effects regression analysis can allow for the prediction of an outcome variable (e.g. hearing loss or age) from a predictor variable (e.g. noise exposure) over time by using repeated measures (Field, 2009). These panel data (repeated observations within an individual) were analysed using a mixed-effect restricted maximum likelihood (REML) regression, for both NEG and NNEG. Because of the many similarities and interactions between NIHL and ARHL, it is imperative to take into consideration the total relevant contribution of ARHL when determining the effect of noise exposure on hearing (Strauss, Swanepoel, Becker, Eloff, & Hall, 2014). The model therefore adjusted for age, as well as baseline hearing thresholds. The model was also repeated without adjusting for age in order to determine the separate contributions of age and noise on the total hearing loss of the participant over a 4-year period. High-frequency averages of 3 kHz, 4 kHz and 6 kHz (HFA346) and LFA of 0.5 kHz, 1 kHz and 2 kHz (LFA512) were analysed separately. Testing was conducted at the *p* < 0.01 level of significance.

### Ethical considerations

Ethical clearance to conduct the study was obtained from the Research and Ethics Committee of the Faculty of Humanities of the University of Pretoria on 09 September 2014 (Reference no.: 26123445). This study was conducted within the framework of the ethical guidelines as set out in the South African National Health Act (2007) as well as the Guidelines of Practice in the Conduct of Clinical Trials in Human Subjects in South Africa (South African Department of Health, 2000).

## Results

The NEG was on average 6.7 years older than that in NNEG at the time of their first audiogram; therefore, age was corrected for in the original analysis, with the specific aim to separate the effects of noise exposure from the joint effect of noise exposure and ageing. Year-to-year increases in mean hearing thresholds were all statistically significant (*p* < 0.01; Table 2). Mean values for year 1 (baseline year per participant, within 2001–2008) were significantly higher for the NEG than for the NNEG. This was seen in both LFA512 analysis and HFA346, although more prominent for HFA346. Year-o-year increases in mean hearing thresholds were higher for the NEG than for the NNEG when comparing HFA346 but similar when comparing LFA512.

**TABLE 2:** Mixed-effects regression analysis of average hearing over the first four serial (yearly) audiograms for the non-noise exposed group and noise exposed group, adjusted for age.

Frequency	Exposure group	Year	Mean (dB HL)	95% confidence interval	Change from baseline (dB HL)	*p*
HFA346	NEG	1	25.7	25.3–26.1	-	-
		2	28.5	28.2–28.9	2.8	< 0.001
		3	29.0	28.6–29.4	3.3	< 0.001
		4	29.2	28.8–29.6	3.5	< 0.001
HFA346	NNEG	1	18.5	18.1–19.0	-	-
		2	20.9	20.4– 21.3	2.3	< 0.001
		3	21.0	20.5–21.4	2.4	< 0.001
		4	21.4	21.0–21.8	2.9	< 0.001
LFA512	NEG	1	16.1	15.7–16.6	-	-
		2	17.5	17.0–17.9	1.3	< 0.001
		3	17.0	16.6–17.6	1.0	< 0.001
		4	16.8	16.3–17.2	0.6	< 0.001
LFA512	NNEG	1	11.1	10.6–11.5	-	-
		2	12.1	11.6–12.6	1.1	< 0.001
		3	11.8	11.3–12.3	0.8	< 0.001
		4	11.1	11.3–12.3	0.7	< 0.001

HFA, high-frequency average; LFA, low-frequency average; NEG, noise exposed group; NNEG, non-noise exposed group; dB HL, decibels in hearing level.

Additionally, mixed-effects regression results were repeated without correcting for age to represent year-to-year increases in hearing thresholds including noise and age influences. Year-to-year increases in mean hearing thresholds were all statistically significant, with *p* < 0.01 (Table 3). Mean values for year 1 (baseline) were significantly higher for the NEG than for the NNEG. This was seen in both LFA512 and HFA346 analyses, although more prominent for HFA346. Year-to-year increases in mean hearing thresholds were higher for the NEG than for the NNEG in comparing HFA346 but were similar when comparing LFA512. Year-to-year increase in mean hearing thresholds were higher in the analysis where age was uncorrected, identifying age as a significant factor in year-to-year increase in mean hearing thresholds for both exposure groups across LFA512 and HFA346.

**TABLE 3:** Mixed-effects regression analysis of average hearing over the first four serial (yearly) audiograms for the non-noise exposed group and noise exposed group, unadjusted for age.

Frequency	Exposure group	Year	Mean (dB HL)	95% confidence interval	Change from baseline (dB HL)	*p*
HFA346	NEG	1	25.4	25.06–25.82	-	-
		2	28.45	28.08–28.83	3.0	< 0.001
		3	29.1	28.69–29.44	3.6	< 0.001
		4	29.4	29.06–29.81	4.0	< 0.001
HFA346	NNEG	1	18.2	17.8–18.7	-	-
		2	20.8	20.3–21.2	2.6	< 0.001
		3	21.1	20.6–21.6	2.9	< 0.001
		4	21.1	21.3–22.2	3.5	< 0.001
LFA512	NEG	1	15.9	15.4–16.3	-	-
		2	17.4	16.9–17.83	1.5	< 0.001
		3	17.2	16.7–17.6	1.3	< 0.001
		4	17.0	16.5–17.5	1.1	< 0.001
LFA512	NNEG	1	10.9	10.4–11.3	-	-
		2	12.1	11.6–12.5	1.2	< 0.001
		3	11.9	11.4–12.3	1.0	< 0.001
		4	12.0	11.5–12.5	1.1	< 0.001

HFA, high-frequency average; LFA, low-frequency average; NEG, noise exposed group; NNEG, non-noise exposed group; dB HL, decibels in hearing level.

## Discussion

Baseline values from year 1 were significantly higher in the NEG compared to the NNEG group for LFA512 and HFA346. The NEG therefore had damage to their hearing prior to their first audiogram within this set of audiograms. The earliest audiograms within this sample were recorded in 2001, when legislation first made baseline audiograms and annual testing compulsory in South Africa (Republic of South Africa, 2001). Unprotected exposure to these high levels of noise is therefore a probable contributor to the higher baseline values seen in the noise-exposed group.

There was a statistically significant increase in mean hearing thresholds, from year to year, as well as over the 4-year period, in both exposure groups for LFA512 and HFA346. The increase in mean hearing thresholds was however higher for the NEG in both LFA512 and HFA346. Noise is therefore an ongoing and significant factor in employees’ total hearing loss. The classic view of NIHL states that the primary damage areas are the hair cells, and that auditory nerve loss is mostly secondary to hair cell loss. Evidence given by Fernandez et al. (2015) and Liberman (2017) suggests that in both NIHL and ARHL, synaptic connections between the hair cells and cochlear neurons can be destroyed even before hair cell damage occurs.

This challenges the traditional view that the influence of noise on hearing is time limited, implying that the progression of NIHL ceases when noise exposure stops (Fernandez et al., 2015). In our sample, it is therefore possible that even after the introduction of annual testing and noise hygiene programmes in 2001 (Republic of South Africa, 2001), the hearing thresholds of the NEG kept increasing at a faster pace than their NNEG counterparts, which will support a super additive interaction between ageing and noise exposure, following the notion that an already damaged hair cell and hair cells with synaptic loss are more susceptible to further damage from additional factors, such as ageing and noise exposure.

A second probability of the increase over time of the NEG hearing thresholds can possibly be attributed to findings by Basner et al. (2014), indicating that despite the introduction of standards for hearing protection, hearing loss because of occupational noise remains a problem. Many countries enforce health and safety legislations pertaining to noise exposure; however, for legislation to work effectively, strict adherence should be enforced. In a study investigating hearing protection device usage in South Africa, the observed use of hearing protection devices (50%) was much lower than the reported use (93%) (Hansia & Dickinson, 2010). Similar results were obtained by Kanji, Khoza-Shangase and Ntlakana (2018) who reported less than 50% consistent use of hearing protection devices. These reports are concerning as evidence exists that even a single synoptopathic exposure can accelerate cochlear ageing (Fernandez et al., 2015). In its simplest form, hearing conservation programmes should be an effective strategy in the management of occupational NIHL. However, current literature like that quoted above indicates that hearing conservation programmes are not achieving the anticipated outcomes within the South African mining sector. This is despite the efforts focussed on the management of NIHL (Moroe, Khoza-Shangase, Madahona, & Nyandoro, 2019). It is therefore possible that our study population is still exposed to occupational noise, even with legislated hearing conservation programmes. One possible contributor to the lack of progress towards the elimination of occupational NIHL may be the fact that occupational audiologists are only marginally involved in the development and implementation of hearing conservation programmes (Moroe & Khoza-Shangase, 2018). Kanji et al. (2018) recommend that the occupational audiologists in South African mines should play an important role in individualised education during audiological testing in order to enhance the effectiveness of hearing conservation programmes. Comprehensive education and training programmes regarding noise exposure and noise measurements with consistent hearing protective device use are needed. A need therefore exists for the mining industry to re-focus its energy on new and innovative ways of understanding why certain components of hearing conservation programmes are not yielding the positive outcomes expected (Moroe, 2018).

Although noise exposure tends to be classified as a predominantly high-frequency hearing loss (Dias et al., 2006; Teles & Medeiros, 2007), the difference between mean hearing thresholds in the NEG and NNEG in this sample was noticed in both the low frequencies (LFA512) and high frequencies (HFA346), although more prominent in the high frequencies. A possible explanation of the increase in low-frequency thresholds in the NEG can be found in a study conducted by Fernandez et al. (2015). In this study, permanent versus non-permanent synaptic loss between hair cells and auditory nerve fibres in mice was compared as they aged. As exposed adult mice aged, synoptopathy was exacerbated compared with controls, and over time, damage spread from the high frequencies to the lower frequencies, which correlates with the findings in the current study.

When the analysis was repeated without correcting for age (in order to get a view of the combined effect of noise and age), there was a marked difference in mean hearing thresholds in both exposure groups when compared with the analysis where age was corrected for. This was observed for both LFA512 and HFA346. The relative contribution of age to the total hearing loss was similar when comparing LFA512 between exposure groups, but more dominant in the NEG when comparing HFA346. Age is therefore a significant contributor, although more prominent in the high frequencies, and the NEG.

The mean age for the first test was higher in the NEG (43.3 years) as opposed to the NNEG (36.6 years), which may act as a moderating factor in the sense that the older the participant, because of the effect of ageing on the ear, the higher their initial hearing thresholds will be. This finding can also be compared to findings that suggest pathological changes from early noise exposure to substantially increase the risk of inner ear ageing and related hearing loss in later years (Campo et al., 2011; Gates et al., 2000; Kujawa & Liberman, 2006; Meneses-Barriviera, Melo, De Moraes, & Marchiori, 2013). Although we cannot accurately identify the specific mechanism for the accelerated increase in hearing thresholds seen in this analysis, it seems possible that the noise-damaged ear does not age at the same rate as an ear without known noise damage; this again points to a possible super additive interaction between NIHL and ARHL. As previously discussed, this is supported by recent evidence by Fernandez et al. (2015) and Liberman (2017) that both noise exposure (even noise exposure only causing TTS) and ageing cause early synaptic damage, which will not be picked up by a standard audiogram, but will form a basis of damage that will greatly influence the progression of hearing loss in later years.

This calls into question assumptions that have been made on the relative contributions of ARHL and NIHL in cross-sectional analysis (Gates et al., 2000). Cross-sectional data can overestimate the relevant contribution of age because the accelerated time-related degeneration would be attributed only to age, rather than to previous noise damage. Gates et al. (2000) supported this notion from a study looking at longitudinal threshold changes in older men. In a cross-sectional study of 40 123 gold miners in South Africa, employing the same data as the current study, it was found that age was the most important influence on hearing thresholds, for both noise and non-noise exposed mine workers (Strauss et al., 2014). It is therefore possible that this conclusion could overstate the relative contribution of age, as in its cross-sectional nature, the accelerated time-related degeneration as an interaction between noise and age was not taken into consideration. It should however also be noted that the mentioned study used a much larger cohort of the total data set (40 123 mine employees) compared to the current study (2536 mine employees). Caution should therefore be exercised when making direct comparisons between the two studies.

While noise exposure and ageing can be seen as the main contributors in the total increase in average hearing thresholds over time for both exposure groups in this sample, caution should however be exercised to disregard other possible contributions to the increase in hearing thresholds in these groups. Hearing loss in the sample should be seen as multifactorial, with contributions from numerous intrinsic and extrinsic variables (Kujawa & Liberman, 2006; Yamasoba et al., 2013). Certain factors that may affect the audiological outcome of the study participants, such as genetic predisposition to hearing loss, health co-morbidities (such as TB, HIV and/or ototoxicity) and environmental influences (such as non-occupational noise exposure or chemical exposure) on hearing, could not be considered. The participants in this study are all from the same occupational background, with similar socio-economic status, living conditions, health exposures and leisure activities; therefore, comparisons between noise exposure groups in this study could be made without fear of one group having substantial other hearing risks above a second group.

## Conclusion

Both age and occupational noise exposure influence hearing thresholds significantly over time, even in a setting where noise exposure is supposed to be controlled through legislation. The continued increase in hearing thresholds of the NEG above that of the NNEG can either be related to ineffective noise-management programme implementation and/or support the fact that early noise exposure leads to a higher burden of hearing loss in later life, even after noise exposure is stopped.
