# Corrigendum: Occupational noise and age: A longitudinal study of hearing sensitivity as a function of noise exposure and age in South African gold mine workers

**DOI:** 10.4102/sajcd.v68i1.849

**Published:** 2021-11-08

**Authors:** Leoni M. Grobler, De Wet Swanepoel, Susan Strauss, Piet Becker, Zahan Eloff

**Affiliations:** 1 Department of Speech-Language Pathology and Audiology, Faculty of Humanities, University of Pretoria, Pretoria, South Africa; 2 Department of Health Sciences, Faculty of Health, University of Pretoria, Pretoria, South Africa; 3 Occupational Health Department, AngloGold Ashanti, Carletonville, South Africa

In the published article, Grobler, L.M., Swanepoel, D.W., Strauss, S., Becker, P., & Eloff, Z. (2020). Occupational noise and age: A longitudinal study of hearing sensitivity as a function of noise exposure and age in South African gold mine workers. *South African Journal of Communication Disorders, 67*(2), a687. https://doi.org/10.4102/sajcd.v67i2.687, there was an error in the affiliations. Instead of:

‘**Authors:**

Leoni M. Grobler^1^

De Wet Swanepoel^2^

Susan Strauss^2^

Piet Becker^3^

Zahan Eloff^4^

**Affiliations:**

^1^Department of Speech-Language Pathology and Audiology, Faculty of Humanities, University of Pretoria, Pretoria, South Africa

^2^Department of Speech-Language Therapy and Audiology, Faculty of Humanities, University of Pretoria, Pretoria, South Africa

^3^Department of Health Sciences, Faculty of Health, University of Pretoria, Pretoria, South Africa

^4^Occupational Health Department, AngloGold Ashanti, Carletonville, South Africa’,

it should be:

‘**Authors:**

Leoni M. Grobler^1^

De Wet Swanepoel^1^

Susan Strauss^1^

Piet Becker^2^

Zahan Eloff^3^

**Affiliations:**

^1^Department of Speech-Language Pathology and Audiology, Faculty of Humanities, University of Pretoria, Pretoria, South Africa

^2^Department of Health Sciences, Faculty of Health, University of Pretoria, Pretoria, South Africa

^3^Occupational Health Department, AngloGold Ashanti, Carletonville, South Africa’.

The authors apologise for this error. The correction does not change the study’s findings of significance or overall interpretation of the study’s results or the scientific conclusions of the article in any way.

